# Clinical outcomes in the era of test and treat among children living with HIV: a retrospective before/after study in Zambia

**DOI:** 10.3389/fped.2025.1480705

**Published:** 2025-10-24

**Authors:** Benson M. Hamooya, Simon Mutembo, Lukundo Siame, Matenge Mutalange, Chilala Cheelo, Kingsley Kamvuma, Brian Muyunda, Keith Mweebo, Nzali Kancheya, Callistus Kaayunga, Morgan Sakala, Johanzi Mvula, Salazeh Kunda, Shem Kabesha, Clive Banda, Derrick Sikaulu, Isaac Fwemba, Sepiso K. Masenga

**Affiliations:** ^1^ School of Medicine and Health Sciences, Mulungushi University, Livingstone, Zambia; ^2^ International Vaccine Access Center, Department of International Health, Bloomberg School of Public Health, Johns Hopkins University, Baltimore, MD, United States; ^3^ Centers for Disease Control and Prevention, DGHT, Lusaka, Zambia; ^4^ Provincial Health Office, Ministry of Health, Choma; ^5^ School of Medicine, University of Zambia, Lusaka, Zambia

**Keywords:** retention, HIV, testing, antiretroviral therapy, Zambia, clinical outcomes, children

## Abstract

**Background:**

Initiating antiretroviral therapy (ART) immediately after diagnosis of HIV infection may reduce morbidity and mortality in children living with HIV (CLHIV), especially when they are retained in HIV care. In Zambia, the retention rate of CLHIV was unknown. The goal of this study was to determine the retention rate and clinical outcomes of implementing a test-and-treat program in children diagnosed with HIV infection.

**Methods:**

We conducted a retrospective before/after study in 42 health facilities in 12 districts of the southern region of Zambia. We reviewed case files of CLHIV initiated on ART before the test-and-treat (BTT) policy (1 January 2014 to 31 July 2016; *n* = 405) and after implementation of the test-and-treat (ATT) policy (1 August 2016 to 1 October 2020; *n* = 579). We collected demographic, laboratory, and clinical data using a structured data collection form in REDCap. The primary outcome was retention defined as regular attending of appointments or engagement with the ART clinic at 3, 6, 12, 24, and ≥24 months after ART initiation. Descriptive statistics and logistic regression were the statistical methods employed.

**Results:**

The median age of 984 CLHIV was 60 months (interquartile range [IQR] 22–100) and 52.3% (*n* = 515) were girls. The overall retention rate (alive and on treatment) was 82.0% (*n* = 807; 95% confidence interval [CI] 79.5–84.4) after 24 months. A higher proportion of children ATT were retained in care compared to those BTT (91.0% vs. 69.1%; *p* < 0.001). More of the children BTT were transferred out (19.0% vs. 4.8%; *p* < 0.001) and lost to follow-up (11.1% vs. 3.8%; *p* < 0.001) compared to those in the ATT cohort. In addition, there was a significant improvement in the proportion of children in World Health Organization (WHO) clinical stage 1, increasing from 83% to 98% among those retained in care at the end of follow-up. In the multivariable analysis, factors associated with higher odds of retention included being in the ATT cohort (adjusted odds ratio [aOR] 4.98; 95% CI 4.06–6.11) and use of a dolutegravir (DTG)-based regimen (aOR 2.66; 95% CI 1.05–6.72). In contrast, being female (aOR 0.80; 95% CI 0.67–0.95), a longer duration from HIV diagnosis to ART initiation (aOR 0.99; 95% CI 0.99–0.99), and being in WHO clinical stage 3 (aOR 0.68; 95% CI 0.52–0.90) or stage 4 (aOR 0.30; 95% CI 0.19–0.48) were negatively associated with retention in care.

**Conclusion:**

Retention was significantly higher among children enrolled in the test-and-treat (ATT) cohort and those on a DTG-based regimen. In contrast, female sex, longer time to ART initiation after HIV diagnosis, and advanced WHO clinical stages were associated with lower odds of retention. However, retention improved as well as clinical outcomes in ATT compared to BTT after 24 months. This study underscores the importance of initiating ART immediately after diagnosis of HIV to enhance retention in HIV care and treatment. Therefore, improving ART retention by enhancing interventions in resource-limited settings can be highly beneficial.

## 1 Introduction

Globally, an estimated 39 million people were living with HIV in 2022, of whom 1.5 million were children aged under 15 years (1). Sub-Saharan Africa (SSA) accounts for 90% of the global burden of HIV among children aged < 15 years (2). To achieve the 95–95–95 targets for children, children living with HIV (CLHIV) must be identified, linked to care, initiated on antiretroviral therapy (ART), and retained in care (3). Retention in care refers to continued engagement in healthcare services after diagnosis, typically measured by attendance at scheduled clinic visits (e.g., at 6, 12, or 24 months after initiation of ART), and serves as a critical indicator of the quality and effectiveness of HIV care programs (4). Without ART, approximately 50% of HIV-infected children die before the age of 2 years and one-third of those who survive past 2 years die before their fifth birthday (5).

Retention of CLHIV in healthcare is critical to prevent HIV-related morbidity and mortality from HIV-related complications and improve viral suppression (6, 7), through ART initiation, monitoring and management of disease progression and treatment failure and provision of medications and supportive care (8). However, retention in HIV care and treatment among children below the age of 15 years is particularly challenging (9). According to the World Health Organization (WHO), sub-Saharan Africa faces a substantial challenge with long-term retention in care (10). Approximately 8%–34% of HIV-infected children are either lost to follow-up, stop treatment, or die (6, 11, 12). Individuals who disengage from care and stop taking medication have a high probability of transmitting HIV infection, progressing to advanced HIV stages (WHO clinical stages 3 and 4), and dying (10). In some cases, only a few children are put on antiretroviral treatment after diagnosis of HIV infection (13) and retention rates at 12 or 24 months can be in the range of 79%–90% (9, 11).

Factors associated with poor retention among children include difficulty accepting HIV status, insufficient human resources and lack of financial resources from healthcare facilities, difficulties attending a clinic during school hours, fear of disclosure to others, social isolation, severe poverty, family conflicts, and negative relationships with healthcare workers (11, 14, 15). After the recommendation by the WHO in 2015–2016 for countries to test and immediately treat HIV infection with antiretroviral therapy, Zambia began implementing this program after August 2016. However, an evaluation of the impact of this implementation on retention in HIV care and clinical outcomes has not yet been evaluated. Although some interventions, like HIV status disclosure, peer support groups, and adherence clubs, have improved retention in care among children and adolescents, others, such as standalone counseling and reminder systems, have had limited or mixed results (16–18). Therefore, we conducted a retrospective before/after study to evaluate the implementation of test and treat in the southern province of Zambia by comparing retention in care for children before the test-and-treat (BTT) and after the test-and-treat (ATT) implementation.

## 2 Methods

### 2.1 Design and population

This was a retrospective before/after study conducted among CLHIV in 42 health facilities from 12 districts of the southern province of Zambia. To allow for before/after analyses, we compared the cohort before (BTT, 1 January 2014 to 31 July 2016) and after (ATT, 1 August 2016 to 1 October 2020) the test-and-treat implementation program. We used programmatic data from the SmartCare system to assess the retention status for each child at 3, 6, 12, 24, and ≥24 months. SmartCare is a Zambia Ministry of Health designated, comprehensive Electronic Health Record in Zambia that is used to collect, store, and report on the aggregate data from the large patient population in a timely manner at facility, district, provincial, and national level. It is a patient-based information system that provides uniquely identifiable data aimed at accurately performing health decisions.

### 2.2 Eligibility

We abstracted the records of children aged up to 14 years who were diagnosed with HIV infection and enrolled on the ART program. We excluded records with missing information on outcome variables and duplicates (identified as repeated entries for the same patient within or across facilities using the unique SmartCare number or national registration number).

### 2.3 Variables in the study

Retention, the primary outcome of this study, was defined as being alive and on treatment, with clinic attendance or engagement at scheduled visits up to 24 months after ART initiation. The independent variables included age, sex, facility location, duration on ART, ART regimen, WHO staging, HIV viral load, attrition factors (transferred out, lost to follow-up [LTFU], and death).

### 2.4 Data collection and sampling

Demographic and clinical data were collected by trained research assistants from the electronic health system (SmartCare) and registers for facilities without SmartCare. Eligible health facilities in each district were listed and assigned unique identification numbers. Using simple random samplings, facilities were selected by generating random numbers in Excel, ensuring equal selection probability. The sample size for each selected facility was then allocated using proportional allocation based on the facility's pediatric ART client load. At the health facility, clients' files were selected using systematic random sampling and each client was followed up through routine clinic visits as recorded in SmartCare or paper-based records and continued until the end of the study or the point of loss to follow-up, transfer, or death. All the data were entered in Research Electronic Data Capture.

### 2.5 Sample size estimation

**Figure 1:**
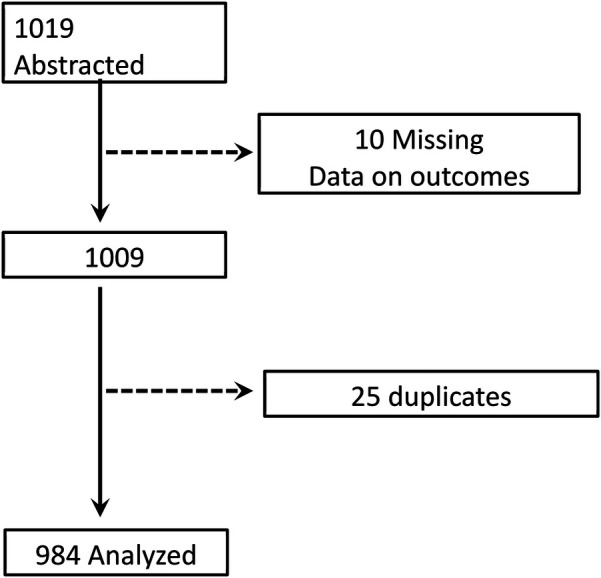
Selection of participants.

The sample size was estimated using the OpenEpi calculator. The prevalence of retention in HIV care before the test-and-treat policy was 85% (19). Assuming a 7% increase in retention (to 92%) following the introduction of the test-and-treat policy, and using a power of 90% with a 5% level of significance, the required sample size was estimated to be 926. At 90% power and a 5% level of significance, we estimated a sample size of 926 and to account for missing information, the sample size was increased to 1,019. However, 984 files were analyzed (Figure 1).

### 2.6 Data analysis

Data were analyzed using Stata version 14.0/IC (Stata Corporation, College Station, TX, USA). Quantitative data were summarized using medians and interquartile ranges (IQR), and qualitative data using frequencies and percentages. The Shapiro–Wilk test was used to determine the normality of the continuous variable. The chi-square and Wilcoxon rank-sum tests were used to ascertain the statistical difference between two categorical variables and two medians, respectively. Adjusted logistic regression (xtlogit model) was fitted to determine factors associated with retention in HIV care and treatment. Variables with a *p*-value <0.2 in the univariate analysis, along with those deemed relevant based on previous literature, were considered for inclusion in the multivariable logistic regression model. A *p*-value <0.05 was considered statistically significant.

### 2.7 Ethical considerations

This study was reviewed and approved by the Macha Research Trust and Zambia National Health Research Authority (ZNHRA) local Institutional Review Boards (IRB) (see 45 C.F.R. part 46. 101(c); 21 C.F.R. part 56). We analyzed de-identified data from health facilities.

## 3 Results

### 3.1 Basic characteristics

We abstracted 984 files of children receiving ART with a median age of 60 months (IQR 22–100). Of them, 52.3% (*n* = 515) were girls. The cohorts were divided into two groups: 405 (41.2%) before the test-and-treat (BTT) policy and 579 (58.8%) after the test-and-treat (ATT) policy. The median age at enrollment was 43 months (IQR 17–76) for the BTT cohort and 73 months (IQR 26–113) for the ATT cohort (*p* < 0.001). Among those retained in care, 48.9% (*n* = 395) were from urban areas and 51.1% (*n* = 416) were from rural areas. The BTT cohort took a median of 5 days (IQR 0–28) to be initiated on ART from the time of HIV diagnosis compared with the ATT cohort, which took 0 days (IQR 0–0; *p* < 0.001). At baseline, of the proportion of participants in WHO stages 2 and 3, ATT was lower compared to BTT (stage 2: 7.8% vs. 10.1%, stage 3: 6.3% vs. 10.6%, and stage 4: 0.4% vs. 3.8%). Conversely, a higher proportion of the ATT participants in comparison to the BTT participants were in WHO stage 1 at baseline (85.5% vs. 75.5%) and currently (98.4% vs. 96.7%; *p* < 0.05). The overall viral suppression at 6 and 12 months was 72.5% (322/444; 95% CI 68.1–76.6) and 81.5% (538/660; 95% CI 78.3–84.4), respectively. Most of the participants who were virally suppressed at 6 months (73.8% vs. 69.9%) and 12 months (83.7% vs. 78.7%) were from the ATT cohort; however, there was no statistical difference. The CD4 count was relatively higher among participants in the cohort BTT compared with the cohort ATT at 6 months (29% vs. 27%; *p* = 0.046) and 12 months (31% vs. 27%; *p* = 0.026) (Table 1).

**Table 1:** Demographic and clinical variables sorted according to cohort status among CLHIV in Zambia between 2014 and 2020.

Characteristic	Before test and treat		After test and treat		*p*-Value
	*n* *=* *405*		*n* *=* *579*		
Age at enrolment (months)^a^	405	43 (17, 76)	579	73 (26, 113)	**<0** **.** **001**
Sex^b^	405		579		0.196
Male		203 (50.1)		266 (45.9)	
Female		202 (49.9)		313 (54.1)	
Facility location^b^	405		579		0.090
Urban		209 (51.6)		267 (46.1)	
Rural		196 (48.4)		312 (53.9)	
Duration to ART initiation^a^		5 (0, 28)		0 (0, 0)	**<0** **.** **001**
Baseline ART-based regimen^b^	401		579		**<0** **.** **001**
NNRTIs (NVP and EFV)		282 (70.3)		282 (48.7)	
INSTI (DTG)		0 (0.0)		38 (6.6)	
PI (LPV/r and ATV/r)		119 (29.7)		259 (44.7)	
Current ART-based regimen^b^	401		578		**<0** **.** **001**
NNRTIs (NVP and EFV)		137 (34.2)		118 (20.4)	
INSTI (DTG)		117 (29.2)		216 (37.4)	
PI (LPV/r and ATV/r)		147 (36.7)		244 (42.2)	
Baseline WHO staging^b^	339		462		**<0** **.** **001**
Stage 1		256 (75.5)		395 (85.5)	
Stage 2		34 (10.1)		36 (7.8)	
Stage 3		36 (10.6)		29 (6.3)	
Stage 4		13 (3.8)		2 (0.4)	
Current WHO staging^b^	393		551		**0** **.** **005**
Stage 1		380 (96.7)		542 (98.4)	
Stage 2		1 (0.3)		6 (1.1)	
Stage 3		8 (2.0)		3 (0.5)	
Stage 4		4 (1.0)		0 (0.0)	
Viral load at 6 months^b^	146		298		0.380
<1,000		102 (69.9)		220 (73.8)	
≥1,000		44 (30.1)		78 (26.2)	
Viral load at 12 months^b^	286		374		0.100
<1,000		225 (78.7)		313 (83.7)	
≥1,000		61 (21.3)		61 (16.3)	
CD4% at baseline^a^	195	24 (15, 32)	177	23 (16, 30)	0.745
CD4% at 6 months^a^	195	29 (21, 42)	168	27 (20, 33)	**0** **.** **046**
CD4% at 12 months^a^	200	31 (25, 36)	138	27 (22, 34)	**0** **.** **026**

^a^ Data are presented as *M* (Q_1_, Q_3_).

^b^ Data are presented as *n* (%).

*n*, number of no-missing values; ART, antiretroviral therapy; M (Q_1_, Q_3_), median (lower quartile, upper quartile); *n* (%), frequency and percentage; CLHIV, children living with HIV; NNRTI, non-nucleoside/nucleotide reverse transcriptase inhibitor; EFV, efavirenz; NVP, nevirapine; PI, protease inhibitor; LPV/r, lopinavir/ritonavir; ATV/r, atazanavir/ritonavir; INSTI, integrase strand transfer inhibitor; DTG, dolutegravir; WHO, World Health Organization.

Bold values indicate statistically significant results (*p* < 0.05).

### 3.2 Follow-up outcomes

Table 2 shows the treatment outcomes by cohort. Overall retention (alive and on treatment) was 82.0% (807/984; 95% CI 79.5–84.4). A higher proportion of children in the ATT cohort were retained in care compared to those in the BTT cohort (91.0% vs. 69.1%; *p* < 0.001). More children in the BTT cohort compared to the ATT cohort were transferred out (19.0% vs. 4.8%) and lost to follow-up (11.1% vs. 3.8%).

**Table 2:** Treatment outcomes by cohort among CLHIV in Zambia between 2014 and 2020.

Treatment outcomes		Before test and treat	After test and treat	*p*-Value
	Overall	*n* = 405	*n* = 579	
Retained in HIV care, yes *n* (%)	807 (82.0)	280 (69.1)	527 (91.0)	<0.001
Stopped treatment, yes *n* (%)	1 (0.1)	0 (0.0)	1 (0.2)	1.00
Transferred out, yes *n* (%)	105 (10.7)	77 (19.0)	28 (4.8)	<0.001
Lost to follow-up, yes *n* (%)	67 (6.8)	45 (11.1)	22 (3.8)	<0.001
Died, yes *n* (%)	4 (0.4)	3 (0.7)	1 (0.2)	0.312

*n* (%), frequency and percentage, respectively; CLHIV, children living with HIV.

### 3.3 Retention by different time points

**Figure 2:**
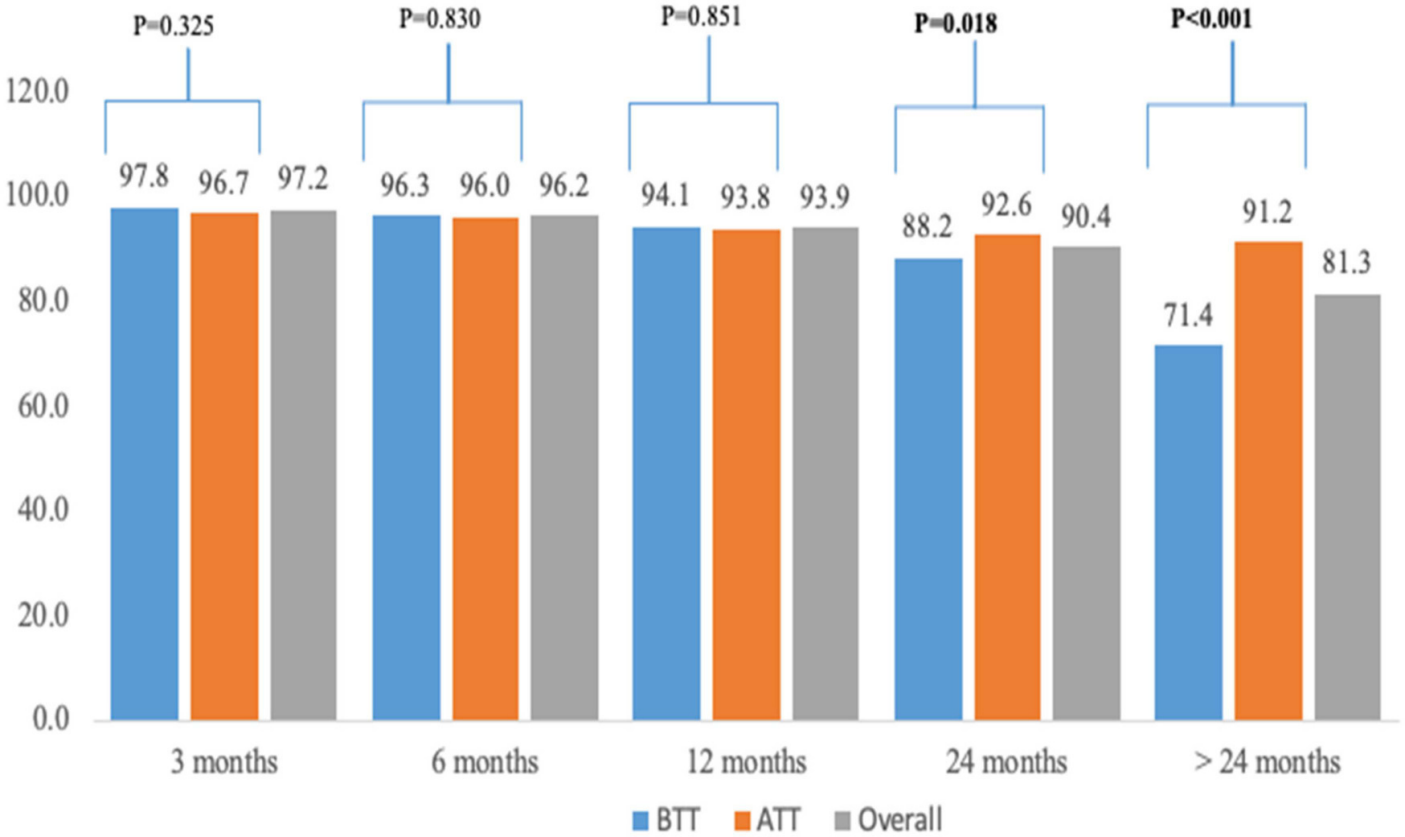
Retention by different time points among CVHIV in Xambia between 2014 and 2020. The *p*-value compares the BTT and ATT cohort.

Figure 2 shows retention at different time points segregated by ATT and BTT policies. At 24 months (92.6% vs. 88.2%) and >24 months (91.2% vs. 71.4%), a significantly higher proportion of children in the ATT cohort were retained in HIV treatment and care compared to those in the BTT cohort (*p* < 0.05). Retention rates at 3, 6, and 12 months were similar between the two cohorts, with no statistically significant differences observed.

### 3.4 Relationship between retention and study covariates

Table 3 shows the relationship between retention in HIV care and other study characteristics. The median age of the retained participants was significantly higher than the ones who were not retained in HIV care (61 vs. 51 months; *p* = 0.009). A greater proportion of the retained CLHIV were from the ATT cohort: 65.3% (*n* = 527) vs. 34.7% (*n* = 280). A higher proportion of the participants retained in HIV treatment and care were on a dolutegravir (DTG)-based regimen at baseline (4.6% vs. 0.6%; *p* = 0.024) and currently (40% vs. vs. 6.8%; *p* < 0.001). Most participants in baseline (83% vs. 73.8%; *p* = 0.001) and current (98.6% vs. 93.3%; *p* < 0.001) WHO staging 1 were retained in HIV care in comparison to unretained ones. Children who were retained in HIV care had higher baseline weights compared with those who were not retained (16 kg vs. 15 kg; *p* = 0.001).

**Table 3:** Relationship between retention and other study variables among CLHIV in Zambia between 2014 and 2020.

Variable	*n* = 984	Retention in HIV care		*p*-Value
		Yes, 807 (82.0)	No, 177 (18.0)	
Age at enrollment (months)^a^	984	61 (23, 103)	51 (16,84)	**0** **.** **009**
Cohort^b^	984			**<0** **.** **001**
BTT		280 (34.7)	125 (70.6)	
ATT		527 (65.3)	52 (29.4)	
Sex^b^	984			0.290
Male		391 (48.5)	78 (44.1)	
Female		416 (51.5)	99 (55.9)	
Facility location^b^	984			0.443
Urban		395 (48.9)	81 (45.8)	
Rural		412 (51.1)	96 (54.2)	
Baseline ART based regimen^b^	980			**0** **.** **024**
NNRTIs (NVP and EFV)		453 (56.3)	111 (63.1)	
INSTI (DTG)		37 (4.6)	1 (0.6)	
PI (LPV/r and ATV/r)		314 (39.1)	64 (36.3)	
Current ART based regimen^b^	979			**<0** **.** **001**
NNRTIs (NVP and EFV)		161 (20.0)	94 (53.4)	
INSTI (DTG)		321 (40.0)	12 (6.8)	
PI (LPV/r and ATV/r)		321 (40.0)	70 (39.8)	
Duration to ART initiation^a^, days^c^	984	0 (0, 9)	0 (0, 22)	**0** **.** **001**
Baseline WHO staging^b^	801			**0** **.** **001**
Stage 1		541 (83.0)	110 (73.8)	
Stage 2		57 (8.7)	13 (8.7)	
Stage 3		47 (7.2)	18 (12.1)	
Stage 4		7 (1.1)	8 (5.4)	
Current WHO staging^b^	944			**<0** **.** **001**
Stage 1		769 (98.6)	153 (93.3)	
Stage 2		6 (0.8)	1 (0.6)	
Stage 3		5 (0.6)	6 (3.7)	
Stage 4		0 (0.0)	4 (2.4)	
Baseline weight (kg)^a^	926	16 (10.5, 23)	15 (9, 19)	**0** **.** **001**

^a^ Data are presented as *M* (Q1, Q3).

^b^ Data are presented as *n* (%).

^c^ Period between enrolment and ART initiation in days.

*n*, number of no-missing values; ART, antiretroviral therapy; *M* (Q_1_, Q_3_), median (lower quartile, upper quartile); *n* (%), frequency and percentage; CLHIV, children living with HIV; NNRTI, non-nucleoside/nucleotide reverse transcriptase inhibitor; EFV, efavirenz; NVP, nevirapine; PI, protease inhibitor; LPV/r, lopinavir/ritonavir; ATV/r, atazanavir/ritonavir; INSTI, integrase strand transfer inhibitor; DTG, dolutegravir; WHO, World Health Organization; BTT, before test and treat; ATT, after test and treat; kg, kilogram.

Bold values indicate statistically significant results (*p* < 0.05).

### 3.5 Logistic regression analysis of factors associated with retention

Table 4 shows the factors associated with retention. At crude analysis, increasing age was associated with a higher likelihood of retention in HIV treatment and care (*p* < 0.001). Children in the ATT cohort had a greater chance of being retained in HIV care compared with those in the BTT cohort (*p* < 0.001). Girls were less likely to be retained than boys (*p* = 0.015). Being on a DTG-based regimen and protease inhibitors (PI)-based ART regimen was associated with a higher likelihood of being retained in HIV care (*p* < 0.001**)**. A longer duration between HIV diagnosis and ART initiation was significantly associated with a lower chance of being retained in HIV treatment and care (*p* < 0.001). Children with advanced WHO clinical stages (stages 3 and 4) were significantly less likely to be retained compared to those in stage 1 (*p* < 0.001). However, in the adjusted analysis, the factors significantly associated with retention were connected with the ATT cohort, where children had a higher chance of being retained in HIV care than those in the BTT cohort (*p* < 0.001). Girls had a reduced likelihood of being retained in HIV care compared with boys (*p* = 0.013). Children on DTG-based ART regimen had a higher chance of being retained in HIV care than those receiving non-nucleoside/nucleotide reverse transcriptase inhibitor (NNRTI) (*p* = 0.038). A longer duration between the time of HIV diagnosis to ART initiation was associated with less chance of being retained in HIV care (*p* < 0.001). Participants in WHO stages 3 and 4 also had a lower chance of being retained in HIV treatment and care (*p* < 0.05).

**Table 4:** Crude and adjusted factors associated with retention among CLHIV in Zambia between 2014 and 2020.

Characteristic	Crude analysis		Adjusted analysis	
	cOR (95% CI)	*p*-Value	aOR (95% CI)	*p*-Value
Age at enrollment (months)	1.01 (1.004, 1.01)	**<0** **.** **001**	0.99 (0.99–1.00)	0.888
Cohort				
BTT	Ref		Ref	
ATT	5.26 (4.44–6.22)	**<0** **.** **001**	4.98 (4.06–6.11)	**<0** **.** **001**
Sex				
Male	Ref		Ref	
Female	0.83 (0.71–0.96)	**0** **.** **015**	0.80 (0.67–0.95)	**0** **.** **013**
Facility location				
Urban				
Rural	0.87 (0.74–1.01)	0.059	0.93 (0.78–1.11)	0.446
Baseline ART based regimen				
NNRTIs (NVP and EFV)	Ref		Ref	
INSTI (DTG)	8.13 (3.33–19.89)	**<0** **.** **001**	2.66 (1.05–6.72)	**0** **.** **038**
PI (LPV/r and ATV/r)	1.17 (1.003–1.37)	**0** **.** **046**	0.83 (0.68–1.01)	0.068
Duration to ART initiation, days^a^	0.99 (0.99–0.99)	**<0** **.** **001**	0.99 (0.99–0.99)	**<0** **.** **001**
Baseline WHO staging				
Stage 1	Ref		Ref	
Stage 2	0.86 (0.65–1.14)	0.300	1.00 (0.74–1.35)	0.986
Stage 3	0.57 (0.44–0.75)	**<0** **.** **001**	0.68 (0.52–0.90)	**0** **.** **008**
Stage 4	0.18 (0.110–0.278)	**<0** **.** **001**	0.30 (0.19–0.48)	**<0** **.** **001**

^a^ Period between enrolment and ART initiation in days.

cOR, crude odds ratio; aOR, adjusted odds ratio; BTT, before test and treat; ATT, after test and treat; CI, confidence interval; CLHIV, children living with HIV; Ref, reference group; NNRTI, non-nucleoside/nucleotide reverse transcriptase inhibitor; EFV, efavirenz; NVP, nevirapine; PI, protease inhibitor; LPV/r, lopinavir/ritonavir; ATV/r, atazanavir/ritonavir; INSTI, integrase strand transfer inhibitor; DTG, dolutegravir; *n*, number of no-missing values; ART, antiretroviral therapy; WHO, World Health Organization.

Bold values indicate statistically significant results (*p* < 0.05).

## 4 Discussion

The overall retention rate among the study participants was 82%; however, this was significantly higher in the ATT policy at 24 and >24 months compared to BTT. Factors associated with retention included belonging to the ATT cohort, use of a DTG-based regimen, and early initiation of ART. Girls and children with advanced WHO staging (3 and 4) were less likely to be retained in HIV care.

Our findings suggest that retention and clinical outcomes are better for a cohort initiated on treatment in the test-and-treat era among CLHIV in Zambia. The immediate and long-term positive impact of rapid ART initiation in children cannot be overemphasized as it has been reported in several clinical trials and longitudinal observation studies (7, 11–13, 20, 21). Among the clinical outcomes from our ATT cohort, loss to follow-up, transfers out of the facility, stopped treatment, and death were lower than what most studies have reported in the region (12). This improvement could be attributed to the implementation of the ATT policy aimed at combating attrition, which has improved over time in Zambia.

Our study found a retention rate of over 92% at both 12 and 24 months before and after the test-and-treat policy. This is in contrast with a previous study conducted at Livingstone Central Hospital where they studied 1,039 children aged 0–15 years enrolled between 2003 and 2015. They found that approximately 16% were LTFU, and after tracing 90% of them, they found that 26% had died, 47% defaulted, 13% were continuing ART at other clinics, and 14% had continued treatment with gaps (22). However, it is important to note that these findings represent only 1 of 42 health facilities that we included in our study. Our study also showed generally higher retention compared to a study conducted in 2004–2014 with a larger sample size, which assessed engagement in HIV care and treatment among individuals aged over 5 years in four provinces of Zambia (23). They found that retention in the ART program declined from 70.0% at 12 months and 61.6% at 24 months. However, this was before the test-and-treat program was implemented in Zambia. This suggests that immediate initiation of ART among CLHIV may improve retention and clinical outcomes.

This study found that CLHIV who experienced a delay between diagnosis and initiation of ART were less likely to be retained in care. Although few studies have directly examined this association, research by Patel et al. (2021), Tedaldi et al. (2014), and Abuogi et al. (2016) demonstrate that rapid ART initiation improves viral load suppression and retention in care, as shown in our study (24–27). These findings highlight the importance of scaling up universal treatment for all children with HIV as early as possible to achieve the UNAIDS 95–95–95 goals.

Children on a dolutegravir (DTG)-based ART regimen were more likely to be retained in HIV care and treatment compared to those receiving a NNRTI-based regimen. Although limited research exists specifically on DTG and retention in children, other studies have demonstrated the safety and efficacy of DTG in achieving viral suppression in this population (28–30). This underscores the importance of a DTG-based regimen in ensuring favorable clinical outcomes among CLHIV in low- and middle-income countries. However, more research is warranted to confirm a direct link between DTG-based regimens and improved retention rates in children with HIV.

Children with HIV in advanced WHO stages (WHO stages 3 and 4) were less likely to be retained in care. This aligns with findings from studies in Ethiopia (2022), Eritrea (2022), and Nigeria (2021), which reported poorer retention rates among children in advanced WHO stages (27, 31, 32). High viral load and low CD4 counts associated with advanced HIV can lead to increased susceptibility to opportunistic infections, ultimately resulting in poorer clinical outcomes (33). Therefore, early linkage from diagnosis to treatment can potentially improve a child's WHO stage and contribute to better retention in HIV care and treatment.

Our study reveals a link between being female and having a lower chance of retention in care for children living with HIV. However, research on sex disparities in pediatric HIV care presents conflicting results. Although Jaspan (34) found better treatment outcomes for boys, Munyayi and van Wyk (2023) and Zemariam et al. (2024) reported no significant difference in retention rates by sex (35, 36). These studies suggest that although sex differences may exist in HIV care for children, further research may be needed to fully understand the factors at play.

### 4.1 Strengths and limitations

Our study has several strengths: the large sample size on most variables allows generalizability and has high power. We used a multicenter sample to represent the entire southern region of Zambia. The southern province is one of the provinces with the highest burden of HIV among children in Zambia. Studying this population was therefore advantageous in providing a large-scale effect of the test-and-treat policy. The multicenter model and sampling methods used strengthened the study and minimized bias, providing a comprehensive overview of the whole program in the province. This is one of the studies with a longer follow-up period of 24 months for the BTT and ATT cohorts compared to other similar studies from Africa (11, 13). However, the study had some duplicates and missing data, particularly on the outcome (see Figure 1 for more details). Some of the comparisons were based on a small sample size, which might affect the generalizability of some findings.

## 5 Conclusion

There was a low overall retention rate among CLHIV in Zambia. However, there was a significant improvement after the implementation of the test-and-treat policy after 24 months. Immediate initiation of ART in CLHIV improved retention rates in HIV care. The attrition factors, such as loss to follow-up and transferring out of the facility, remarkably decreased during the period of test-and-treat policy. Our study demonstrates the importance of implementing evidence-based interventions such as the test-and-treat policy to improve the care and survival of children living with HIV.

## Data Availability

The raw data supporting the conclusions of this article will be made available by the authors, without undue reservation.
